# Molecular Genetic Analysis of the Variable Number of Tandem-Repeat Alleles at the Phenylalanine Hydroxylase Gene in Iranian Azeri Turkish Population

**DOI:** 10.7508/ibj.2015.03.009

**Published:** 2015-07

**Authors:** Morteza Bagheri, Isa Abdi Rad, Nima Hosseini Jazani, Rasoul Zarrin, Ahad Ghazavi

**Affiliations:** 1 *Food and Beverages Safety Research Center, Urmia University of Medical Sciences, Urmia, Iran; *; 2 *Cellular and Molecular Research Center, Urmia University of Medical Sciences, Urmia, Iran; *; 3 *Neurophysiology Research Center, Urmia University of Medical Sciences, Urmia, Iran*

**Keywords:** Phenylalanine hydroxylase, Population genetics, Variable numbers of tandem-repeat

## Abstract

**Background:**

The variable numbers of tandem-repeat (VNTR) alleles at the phenylalanine hydroxylase (*PAH*) gene have been used in carrier detection and prenatal diagnosis in phenylketonuria families. This study was carried out to analyze VNTR alleles at the *PAH* gene in Iranian Azeri Turkish population.

**Methods:**

In this study, 200 alleles from general population were studied by PCR.

**Results:**

The frequencies of VNTR alleles were 45%, 46%, 2%, 3%, 1%, and 3% in studied group regarding 3, 8, 9, 11, 12, and 13 repeat copies, respectively. Statistically significant differences were not found between expected and observed frequencies of VNTR genotypes (*P* > 0.05).

**Conclusions:**

VNTR alleles with three and eight repeats were frequent, and the VNTR alleles with 13 repeats showed 3% frequency in the tested group. This study is the first report on tested population genetic structure using VNTR alleles at the *PAH* gene.

## INTRODUCTION

The phenylalanine hydroxylase (PAH) is a hepatic enzyme involved in L-phenylalanine catabolism [1]. A large amount of variations and mutations within *PAH* gene result in defective form of enzyme [1]. Complete or nearly complete deficiency of PAH enzyme is responsible for different phenotypes of hyper-phenylalaninemia and phenyl-ketonuria (PKU) [2]. 

*PAH* gene is located on chromosome 12 (band region q22-24.1) and consists of 13 exons and 12 introns [3]. The human *PAH* gene is 100 kb long and covers 5’ and 3’ un-translated regions [3]. A wide range of (more than 500) disease-causing mutations has been found in human *PAH* gene [4]. In this regard, it is not possible to detect disease-causing mutations by a simple method. 

For prenatal diagnosis and carrier screening of PKU, several linkage analyses may be informative for study such as short tandem repeats (TCTAT)n within intron 3 and multi-allelic variable numbers of tandem-repeat (VNTR), which is a 30-bp AT-rich tandem-repeat system located in 3’ un-translated region of the *PAH *gene [5, 6]. Multi-allelic VNTR at the *PAH* gene could be understood as a simple, rapid and highly informative molecular genetics procedure for diagnosis and carrier screening of PKU [6]. These polymorphic DNA markers have been studied in different populations and showed significant diversity between inter- and intra-geographical regions [7]. A DNA marker may be informative if any randomly selected individual is expected to be heterozygous for that marker, and the informativeness of the markers is one of the most important points in genetic linkage mapping of human chromosomes [8-10]. Importance of marker loci for genetic linkage analysis depends on the alleles and allele frequencies in the population [11]. A marker is known polymorphic if it has at least two alleles [11]. The rate of most common allele in the population is about 99% [12]. Informativeness of polymorphic DNA markers is determined by the calculation of heterozygosity and polymorphism information content (PIC) value, which was used for a codominant marker in a linkage study [12-14]. A proband is defined as informative if we conclude from proband genotype, which marker allele is linked to the disease-causing allele [13, 14]. Marker allele and disease-causing allele are co-inherited in subsequent generations [14]. Iran is defined as one of the most heterogeneous population in the world [9, 15]. Therefore in this study, VNTR alleles at *PAH* gene in an Iranian Azeri Turkish population were analyzed.

## MATERIALS AND METHODS

This study was approved by the ethics committee of Urmia University of Medical Sciences (Urmia, Iran). Between 2012 and 2014, a total number of 100 healthy subjects with age ranging from 20 to 60 years voluntarily entered the study. We studied genetically unrelated healthy subjects who were resident in the West Azerbaijan Province of Iran. All participants were matched for ethnicity and geographical region. Studied individuals were selected in genetic counseling sessions taken place in Genetic Center of Motahari Hospital (Urmia). All healthy subjects were chosen randomly regarding their past medical history and exclusion of specific findings. A written informed consent was obtained from the studied subjects. DNA was isolated from blood samples using a ‘salting out’ method [16]. Analysis of the genomic DNA was carried out by PCR using the 5’-ttt taa tgt tct cac ccg cc-3’ and 5’-aag aat ccc atc tct cag ag-3’ primers [5]. The PCR conditions on the thermal cycler (Eppendorf) was as follows: an initial denaturation step at 95°C for 5 min, followed by 35 cycles of 1 min at 95°C, 1 min at 55°C, 1 min at 72°C, and a final extension step of 5 min at 72°C. Amplified PCR fragments were separated on 2% agarose gel and stained with ethidium bromide. Presence or absence of different amplified DNA fragments, which are responsible for different alleles, were visualized under UV light. Electrophoretic analysis of the amplified PCR products verified six distinct sizes 325, 475, 505, 565, 595, and 625 bp. These size differences reflected the presence of 3, 8, 9, 11, 12, and 13 copies of the repeated unit, respectively. 

***Statistical analysis. ***The frequencies of VNTR alleles and genotypes were determined via direct counting and dividing by the number of chromosomes and the number of subjects. The Hardy-Weinberg equilibrium (HWE) test for multiple VNTR alleles were carried out regarding equation (p + q + r + s + t + u)^2^ = 1 at a locus. In this study we used runs of homozygosity (ROH), heterozygosity (H), and PIC value to detect the informativeness of polymorphic VNTR alleles. ROH [17] and H [18] values at the PAH locus in the studied population were calculated by the following equations:

$\mathrm{ROH}=\sum_{i=1}^{n}{\mathrm{Pi}}^{2}=p^{2}+q^{2}+r^{2}+s^{2}+t^{2}+u^{2}$ = 

$H=1-\mathrm{ROH}=1-\sum_{i=1}^{n}{\mathrm{Pi}}^{2}$

PIC value was determined via formula (12):

$\mathrm{PIC}=1-\sum_{i}^{n}{\mathrm{Pi}}^{2}-2\sum_{i=1}^{n-1}\sum_{j=i+1}^{n}{\mathrm{Pi}}^{2}{\mathrm{Pj}}^{2}=1-\sum_{i}^{n}{\mathrm{Pi}}^{2}-$ ${\left(\sum_{i=1}^{n}{\mathrm{Pi}}^{2}\right)}^{2}$+$\sum_{i=1}^{n}{\mathrm{Pi}}^{4}$

Where Pi and Pj are the population frequency of the i^th^ and j^th^ alleles, n= number of different alleles at single locus. Degree of freedom, the chi-square (χ^2^) distribution, χ^2^ critical value, and *P* value were analyzed for testing the agreement between the observed and expected frequencies of VNTR genotypes. Microsoft Office Excel 2010 was used for statistical analysis. χ^2^ critical value was found from χ^2 ^table based on degree of freedom and α = 0.05. Significance level was accepted at *P* value less than 0.05.

## RESULTS

**Fig. 1:**
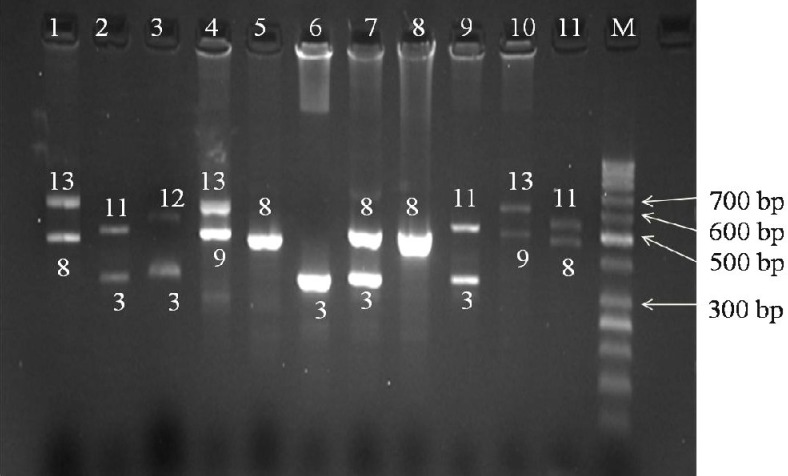
Identification of VNTR alleles in 11 samples by PCR test in Iranian Azeri Turkish general population. PCR yields fragments of 325, 475, 505, 565, 595, and 625 bp containing 3, 8, 9, 11, 12, and 13 repeat copies, respectively. Lane 1, 8/13; Lane 2, 3/11; Lane 3, 3/12; Lane 4, 9/13; Lane 5, 8/8; Lane 6, 3/3; Lane 7, 3/8; Lane 8, 8/8; Lane 9, 3/11; Lane 10, 9/13; Lane 11, 8/11; M: 50 bp DNA ladder.

In this investigation, 200 alleles were studied in a general population. Number and size range of VNTR alleles, HWE, ROH, H, and PIC values were also analyzed. The results of this study are summarized in Table 1. Of 200 chromosomes, 6 VNTR alleles were identified at the *PAH* gene. The PCR products were six alleles of 325, 475, 505, 565, 595, and 625 bp containing 3, 8, 9, 11, 12, and 13 repeat copies, respectively. The frequencies of VNTR alleles at the *PAH* gene were 90 (45%), 92 (46%), 4 (2%), 6 (3%), 2 (1%), and 6 (3%) in studied group regarding 3, 8, 9, 11, 12, and 13 repeat copies, respectively. VNTR 5, 6, 7, and 10 alleles were not found in our study. The frequency of VNTR genotypes meets HWE, and the analysis of the χ^2^ distribution showed that there is an agreement between the observed and those of expected frequencies (χ^2 ^ = 2.33E-20 < 31.41 (χ^2 ^CV), α = 0.05, degree of freedom = 20, *P* = 1). The findings of this study showed that the observed ROH and expected ROH were 0.146 and 0.416, demonstrating that the observed H and expected H values were 0.854 and 0.583, respectively. No statistically significant differences were found between observed H and expected H (χ^2^ = 0.18 < 3.84, *P* = 0.67). By calculation of PIC value using the formula for PIC, we found that PIC value for VNTR alleles at the *PAH* gene equals 0.496. Electrophoretic analysis of different VNTR alleles at the *PAH* gene, including 3, 8, 9, 11, 12, and 13 copies of the repeated units in 11 samples are shown in Figure 1. 

**Table 1:** Variable numbers of tandem-repeat genotypes in Iranian Azeri Turkish population

**No.**	**Genotype**	**Observed**	**% Observed**	**Expected**	**% Expected**
1	3,3	26	0.26	0.2025	20.25
2	3,8	32	0.32	0.4140	41.40
3	3,11	4	0.04	0.0270	2.70
4	3,12	2	0.02	0.009	0.90
5	8,8	28	0.28	0.2116	21.16
6	8,11	2	0.02	0.0276	2.76
7	8,13	2	0.02	0.0276	2.76
8	9,13	4	0.04	0.0012	0.12
9	9,3	0	0	0.0180	1.80
10	13,3	0	0	0.0270	2.70
11	8,9	0	0	0.0184	1.84
12	8,12	0	0	0.0092	0.92
13	9,9	0	0	0.0004	0.04
14	9,11	0	0	0.0012	0.12
15	9,12	0	0	0.0004	0.04
16	11,11	0	0	0.0009	0.09
17	11,12	0	0	0.0006	0.06
18	11,13	0	0	0.0018	0.18
19	12,12	0	0	0.0001	0.01
20	12,13	0	0	0.0006	0.06
21	13,13	0	0	0.0009	0.09

## DISCUSSION

The frequencies of multi-allelic VNTR at the *PAH* gene is significantly different among ethnic groups [19]. A total of six VNTR alleles at the *PAH* gene were recognized. The frequencies of VNTR were 45%, 46%, 2%, 3%, 1%, and 3% at the *PAH* gene in Iranian Azeri Turkish population regarding 3, 8, 9, 11, 12, and 13 repeat copies, respectively. In genetic loci, including multiple alleles (more than 10), the number of genotype(s) is/are large and some of the other genotypes are absent. VNTR 5, 6, 7, and 10 were also absent in this study. The VNTR allele with three and eight repeat copies had the highest frequency in the studied group. In agreement with other studies [15, 20, 21], we here reported the VNTR alleles with 13 copies in Iranian Azeri Turkish population (West Azerbaijan Province of Iran). Interestingly, the VNTR alleles with 13 copies at the PAH locus are specific to Iranian population [15, 20, 21]. Our findings implied that VNTR alleles at the *PAH* gene are known to be moderately informative [22]. The high rate of heterozygosity at the PAH earns a great number of genetic variability. In this investigation, the observed heterozygosity was 0.854. This high rate of heterozygosity was consistent to Fazeli and Vallian (70%) [21] and Kamkar *et al.* (66%) [15]. These findings demonstrate a high degree of heterozygosity for the VNTR alleles at the *PAH* gene in Iranian population. Considering random mating, PIC value for VNTR alleles at the *PAH* gene was 49.6% in this study. PIC value was also 70%, 32%, and 66% in European Caucasians, Chinese, and Iranian [23, 24], respectively. Highly informative level of PIC is set at PIC value ≥ 0.7 [22]. At a PIC of 1, the marker would have multiple alleles at a locus [13, 24]. It is clear that markers with higher number of alleles have higher PIC value, and they are considered to be more informative. VNTR alleles in the *PAH* gene was found to be moderately informative in tested group (0.49 vs. 0.70) [22]. The findings of the present study indicate that the degree of heterozygosity for the VNTR markers at the PAH locus was high. 

It can be concluded that the VNTR alleles with three and eight repeats are frequent and the frequency of the VNTR allele with 13 repeats is 3% in Iranian Azeri Turkish population. This study is the first report on tested population genetic structure using VNTR alleles at the *PAH* gene.

The present report is the first in its own kind in tested group and describes population genetic structure using VNTR alleles at the *PAH* gene.
